# Association of uric acid, calcium, and creatinine with bone mineral density in adults over 50: a cross-sectional study

**DOI:** 10.3389/fendo.2026.1772179

**Published:** 2026-03-25

**Authors:** Hongjie Zhang, Jiafang Zhang, Chuxi Wang, Jinze Lin, Senlin Cai, Xianghui Fan, Yingzi Liao, Meiding Liu

**Affiliations:** 1 The Second People’s Hospital Affiliated to Fujian University of Traditional Chinese Medicine, Fuzhou, Fujian, China; 2 Vascular Surgery and Interventional Oncology Center, Shengli Clinical Medical College of Fujian Medical University, Fujian Provincial Hospital, Fuzhou, Fujian, China

**Keywords:** aging, bone mineral density, calcium, creatinine, serum uric acid

## Abstract

**Aims:**

The relationship between uric acid (UA), calcium, creatinine, and bone mineral density (BMD) remains complex and potentially non-linear. Our objective was to explore these associations in individuals aged over 50 years, considering the interactive effects of body mass index (BMI) and renal function.

**Methods:**

Across-sectional study was conducted on 216 patients (aged ≥50 years). BMD was measured by dual-energy X-ray absorptiometry (DXA). Multivariate linear regression, generalized additive models (GAM), and stratified analyses were performed to evaluate the associations between metabolic parameters and BMD.

**Results:**

In multivariate regression, serum creatinine and age were significant predictors of BMD. GAM analyses revealed non-linear relationships: UA showed a positive association with BMD that plateaued at higher levels (>400 umol/L); BMI exhibited an inverted U-shaped association. Stratified analyses indicated that the positive UA-BMD association was significant only in individuals with normal BMI and lower creatinine levels. Feature importance analysis identified UA, BMI, and creatinine as the top predictors of BMD.

**Conclusions:**

In this population, UA, creatinine, and BMI exhibited complex interactive associations with BMD. These findings suggest that the protective effect of uric acid on bone may depend on metabolic status and renal function.

## Introduction

Osteoporosis is a multifactorial condition influenced by genetic, hormonal, and lifestyle factors, as well as oxidative stress (1–3). As a pivotal pathological mechanism, oxidative stress contributes to bone resorption by promoting osteoclast activity an inhibiting osteoblast function (4). In this context, uric acid (UA) has attracted attention as an endogenous antioxidant. Evidence suggests that UA may mitigate free radical damage to bone tissue, thereby protecting bone density (5). Concurrently, calcium remains a pivotal element in bone mineralization, with serum levels showing significant variability associated with fracture risk (6, 7). Elderly individuals frequently experience calcium metabolism disorders due to hormonal changes and diminished absorption, resulting in accelerated bone loss (8). Accordingly, interventions such as calcium and vitamin D supplementation have been shown to yield substantial improvements in bone mineral density (9–11).

The synergistic role of UA and serum calcium in bone metabolism remains to be fully elucidated. While elevated UA may offer protection through antioxidant mechanisms, calcium maintains bone structure by promoting mineral deposition (12, 13).

Specifically, Gómez-de-Tejada-Romero et al. (14) identified a positive correlation between UA and lumbar spine bone density, which was notably more pronounced in cohorts with elevated serum calcium levels. This observation suggests that calcium and UA may exert a concerted influence on bone metabolism. A plausible biological pathway involves the modulation of parathyroid hormone (PTH) secretion by serum calcium (15), which subsequently alters renal uric acid handling and bone turnover rates. Nevertheless, this synergistic effect has been questioned; for instance, Veronese et al. (16) did not identify a significant combined effect of UA and calcium on BMD. Furthermore, serum creatinine, a marker of muscle mass and renal function, shows conflicting associations. In patients with end-stage renal disease, decreased serum creatinine levels are associated with low bone mineral density (17). Conversely, in postmenopausal women, higher serum creatinine levels may be associated with an increased risk of osteoporosis (18).

These divergent findings suggest that the relationship between metabolic markers and BMD varies by population characteristics and requires comprehensive assessment. Based on the background mentioned above, this study aims to explore the correlation between UA and BMD in individuals over 50 years of age, and to analyze the modifying influence of serum calcium. We employed statistical methods including multiple regression, stratified analysis, and nonlinear generalized additive models (GAM) to analyze the clinical data. Specifically, we selected creatinine, uric acid, BMI, age, and calcium as core variables to evaluate their independent and interactive associations with bone mineral density, aiming to provide new insights for the prevention and treatment of osteoporosis.

## Materials and methods

### Study population

A total of 216 patients aged 50 years and above who visited The Second People’s Hospital Affiliated to Fujian University of Traditional Chinese Medicine from October 2022 to October 2024 were included in this study. The study protocol was approved by the Ethics Committee of The Second People’s Hospital Affiliated to Fujian University of Traditional Chinese Medicine (Approval No. 202508–03), and informed consent was obtained from all participants. The study included 216 participants (200 females, 16 males). Gender-stratified analysis was not performed due to the limited male sample size; gender was adjusted for as a covariate.

The inclusion criteria were patients with complete clinical data for uric acid (UA), serum calcium, creatinine, and body mass index (BMI). The exclusion criteria were as follows: (1) missing data for UA, serum calcium, creatinine, or BMI; and (2) a history of vertebroplasty for lumbar compression fractures, as this procedure may artificially elevate bone mineral density (BMD) measurements and confound the results.

### Clinical and biochemical assessments

Basic characteristics, including gender, age, height, weight, body mass index.

(BMI), and medical history, were recorded. Laboratory data were retrospectively collected from the hospital’s biochemical analysis records, including uric acid (UA), calcium, albumin, total cholesterol, triglycerides, creatinine, and phosphorus. Additionally, platelet, neutrophil, and lymphocyte counts were obtained from complete blood count tests.

### Bone mineral density measurement

Bone mineral density (BMD) at the lumbar spine (L1 –L4) was assessed using dual-energy X-ray absorptiometry (DXA) with an EXA-3000 densitometer (OsteoSys).To ensure consistency and minimize measurement error, all scans were performed independently by a single experienced technician following daily quality control procedures.

### Sample size calculation

The sample size was calculated using G*Power software (version 3.1.9.7). Based on a multiple linear regression model designed to evaluate the independent predictive effect of UA on BMD, we assumed a medium effect size (f^2^ = 0.15), a significance level (α) of 0.05, and a statistical power (1-β) of 0.80. These parameters indicated a minimum requirement of participants. The final study population included 216 participants, which exceeded this minimum requirement and ensured sufficient statistical power for the analyses.

### Statistical analysis

Continuous variables were evaluated for normality using the Shapiro –Wilk test. Data with a normal distribution are presented as mean ± standard deviation (SD), while non-normally distributed variables are reported as median with interquartile range (IQR). To satisfy the assumptions of parametric analyses, variables exhibiting skewed distributions—specifically age, height, weight, BMI, L1 –L4 BMD, albumin, total cholesterol, triglycerides, creatinine, neutrophil count, lymphocyte count, and uric acid—underwent natural logarithmic transformation.

Candidate variables for regression models were selected based on correlation coefficients (|r| > 0.2) derived from Pearson and Spearman analyses, with associations visualized using a heatmap. To mitigate multicollinearity, variance inflation factor (VIF) diagnostics were applied, and variables with VIF > 10 were excluded. We compared the goodness-of-fit between multiple linear regression models and non-linear generalized additive models (GAM) to determine the optimal modeling approach. Finally, interaction analyses and feature importance assessments were conducted to elucidate the specific contributions of individual predictors. A two-tailed P < 0.05 was considered statistically significant.

## Result

### Baseline characteristics

The demographic and clinical characteristics of the 216 participants are summarized in **Table 1**.

**Table 1:** Baseline demographic and clinical characteristics N=216.

Variable	N	Mean (SD)	Median [Q1,Q3]
Demographics and anthropometrics			
Age (years)	216	70.38 (9.97)	70.0 [63.0,77.0]
Height (cm)	216	156.80 (5.87)	157.0 [153.0,160.0]
Weight (kg)	216	57.47 (9.32)	57.5 [51.38,62.0]
BMI	216	23.33 (3.37)	23.02 [21.23,25.05]
Bone and Mineral Homeostasis			
Lumbar Spine BMD	216	0.767 (0.141)	0.771 [0.669,0.844]
Calcium (Ca) (mmol/L)	216	2.25 (0.11)	2.26 [2.18,2.31]
Phosphorus (P) (mmol/L)	216	1.16 (0.15)	1.16 [1.07,1.26]
Metabolic and Nutritional Status			
Albumin (ALB) (g/L)	216	41.35 (3.39)	41.8 [39.6,43.3]
Total Cholesterol (TC) (mmol/L)	216	5.19 (1.19)	5.2 [4.4,5.9]
Triglycerides (TG) (mmol/L)	216	1.44 (1.20)	1.2 [0.84,1.62]
Uric Acid (Ua)	216	320.17 (83.47)	305.0 [264.0,365.34]
Renal Function			
Creatinine()	216	60.98 (14.39)	59.0 [53.0,66.0]
Hematologic Features			
Platelet Count (×10^9^/L)	216	229.11 (52.20)	226.0 [195.5,258.25]
Neutrophil Count (×10^9^/L)	216	3.52 (1.42)	3.26 [2.61,4.17]
Lymphocyte Count (×10^9^/L)	216	1.89 (0.59)	1.83 [1.49,2.25]

### Normality transformation and correlation analysis

**Figure 1:**
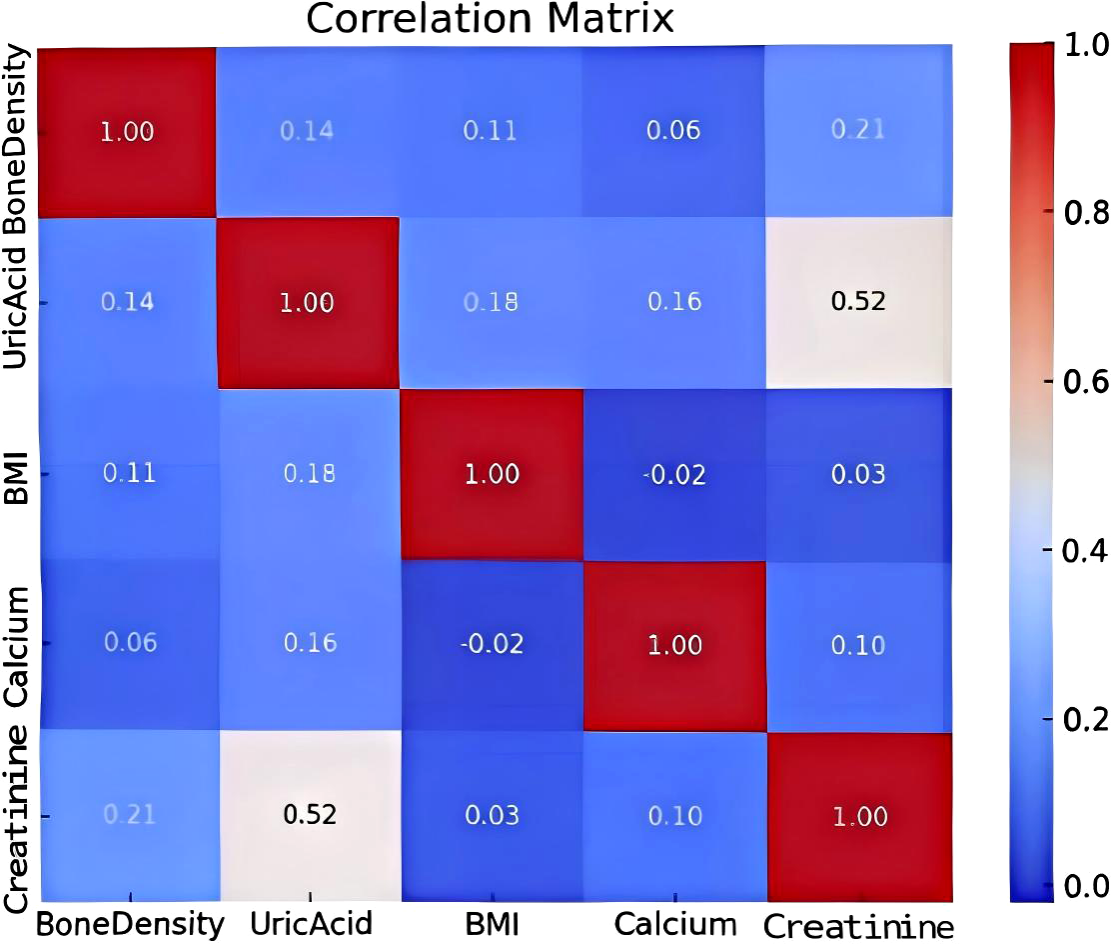
Pearson and Spearman correlation coefficient ciagram.

Correlation analysis and variable selection following normality testing and logarithmic transformation of skewed variables, correlation analyses were performed to identify factors associated with lumbar spine BMD. Both Pearson and Spearman coefficients revealed consistent associations (**Figure 1**).

In the Pearson analysis, serum creatinine (r = 0.207), uric acid (r = 0.144), BMI (r = 0.112), and serum calcium (r = 0.063) exhibited the strongest correlations with BMD. Specifically, creatinine, uric acid, BMI, and calcium all showed positive associations with BMD. The Spearman analysis yielded similar rankings and directions of association (creatinine: r = 0.215, BMI: r = 0.190, uric acid: r = 0.147, calcium: r = 0.058). Based on the magnitude of these correlations (|r|), creatinine, uric acid, BMI, and calcium were selected as candidate variables for subsequent multivariate modeling.

### Multicollinearity test

Assessment of multicollinearity Variance inflation factor (VIF) diagnostics confirmed the absence of significant multicollinearity among the selected independent variables. The VIF values for creatinine (1.38), uric acid (1.48), BMI (1.06), age (1.11), and calcium (1.07) were all substantially below the threshold of 10. Consequently, all candidate variables were included in the subsequent multivariate regression analyses.

### Multivariate linear regression analysis

We constructed four tested linear regression models to evaluate the associations of metabolic markers with BMD (**Table 2**). The models showed statistical significance overall (P < 0.01 for all),with explained variance (R^2^) ranging from 0.045 to 0.078.

**Table 2:** Correlation between metabolic markers and bone mineral density.

Model & predictors	β (coefficient)	P–value	Model R2	Model F-statistic	Model P–value
Model 1			0.045	4.963	0.008
Uric Acid	8.50 × 10−5	0.521			
Creatinine	0.0018	0.022			
Model 2			0.070	4.219	0.001
Uric Acid	0.0001	0.453			
Creatinine	0.0020	0.008			
BMI	0.0031	0.274			
Model 3			0.078	4.280	0.001
Uric Acid	0.0001	0.453			
Creatinine	0.0020	0.008			
BMI	0.0031	0.274			
Age	-0.0028	0.005			
Model 4			0.070	4.219	0.001
Uric Acid	0.0001	0.453			
Creatinine	0.0020	0.008			
BMI	0.0031	0.274			
Age	-0.0028	0.005			
Calcium	0.0036	0.968			

Across all model specifications, serum creatinine demonstrated a consistent and significant positive association with BMD (in Model 4: β = 0.0020, P = 0.008). Age was also identified as a significant negative predictor when included in Models 3 and 4 (β = -0.0028, P = 0.005). However, UA, BMI, and serum calcium did not exhibit statistically significant linear associations with BMD (P > 0.05). Given the lack of significance in linear models despite their physiological relevance, we proceeded to investigate potential non-linear relationships using generalized additive models (GAM).

### Non-linear associations (generalized additive models)

**Figure 2:**
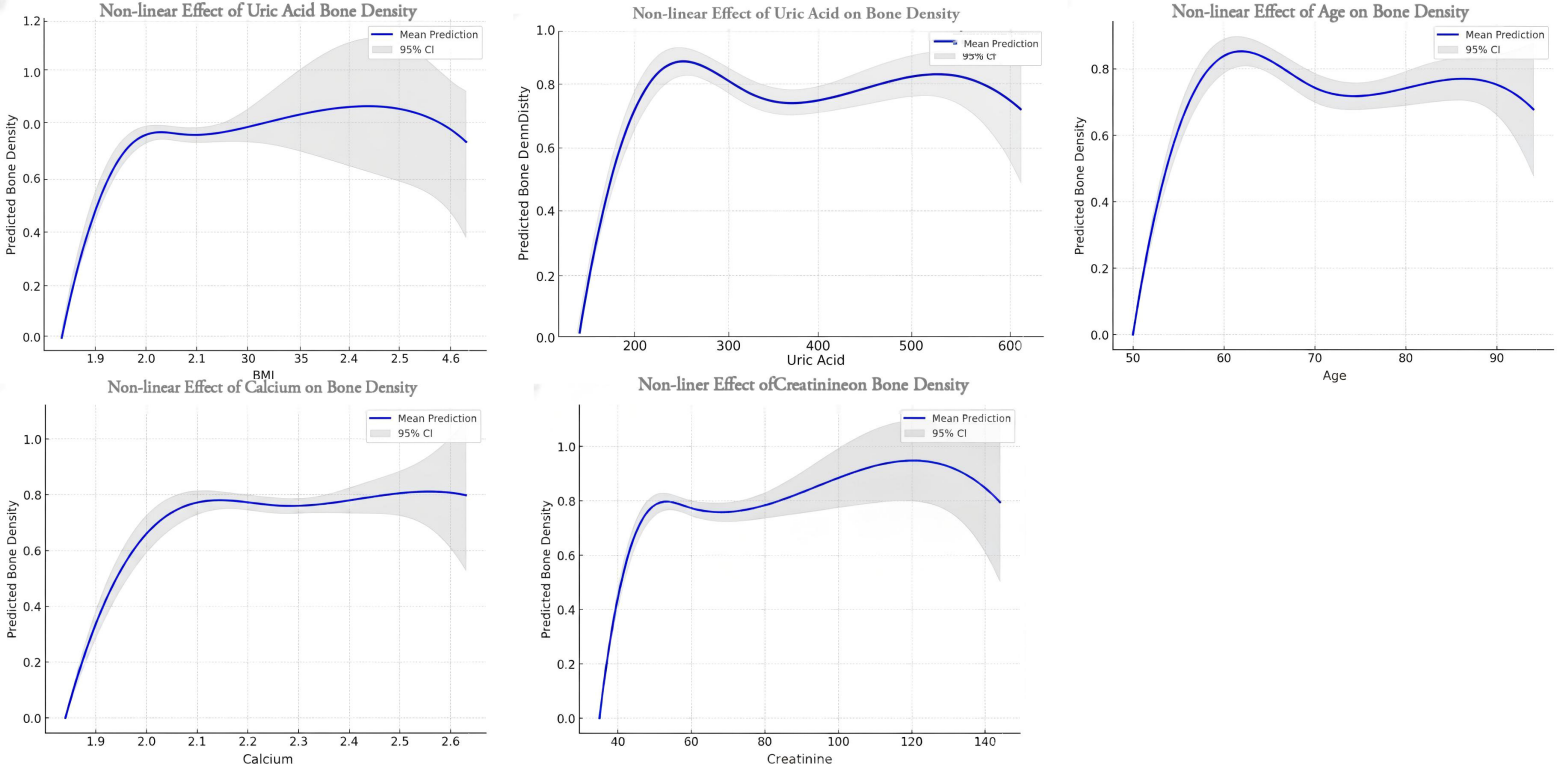
Trend analyses revealed the following non-linear associations.

GAM analyses revealed distinct non-linear patterns in the relationships between clinical variables and BMD (**Figure 2**).

### Metabolic markers (uric acid, calcium, creatinine)

A saturation effect was observed for UA, calcium, and creatinine. Specifically, 168 BMD increased rapidly with UA at lower concentrations (≤200umol/L) but plateaued and exhibited a slight decline at levels exceeding 400umol/L. A similar trajectory was noted for serum calcium, where the positive association observed at low-to-normal levels (2.0–2.5mmol/L) attenuated at concentrations >2.5mmol/L. Likewise, BMD was positively associated with creatinine at lower levels (<80umol/L) but stabilized at moderate-to-high concentrations (>100umol/L).

### Anthropometric and demographic factors (BMI, age)

The relationship between BMI and BMD approximated an inverted U-shaped curve: BMD increased across the normal BMI range (18.5–24.9 kg/m^2^) but flattened or decreased in the overweight and obese range (≥ 25kg/m^2^).Finally, age demonstrated a biphasic trajectory, with BMD remaining relatively stable between 50 and 65 years before declining markedly thereafter.

### Interaction effect analysis

To evaluate potential effect modification, we constructed regression models incorporating interaction terms for UA×calcium, UA×BMI, and UA ×creatinine.

**Figure 3:**
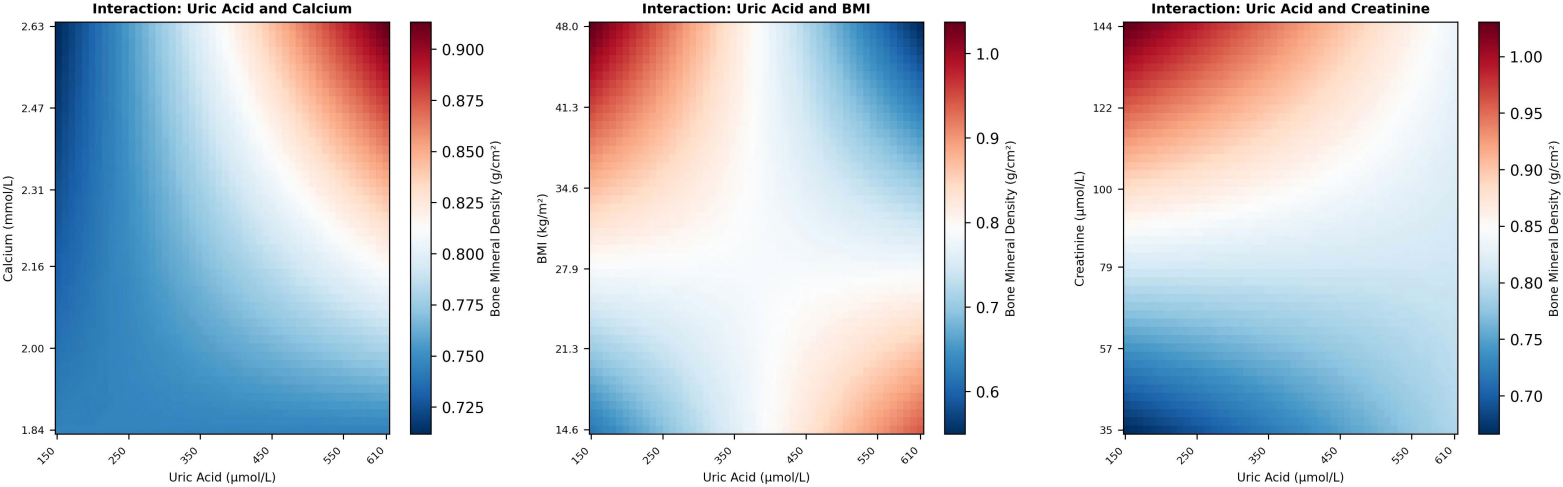
Interaction between uric acid and serum calcium, interaction between uric acid and BMI, and interaction between uric acid and serum creatinine on bone mineral density.

Potential interactions were noted between UA and BMI (P=0.056) and between UA and serum creatinine ( P=0.055), both approaching statistical significance. These results suggest that BMI may modulate the relationship between UA and BMD, indicating that the bone-protective effects of UA are likely weight-dependent. Furthermore, serum creatinine levels appeared to modify the UA-BMD relationship, with elevated creatinine concentrations tending to attenuate the positive association observed at lower levels. In contrast, no interaction was observed between UA and serum calcium ( P=0.530) (**Figure 3**).

Based on these findings, stratified analyses were performed to further characterize the relationships across different subgroups.

### Stratified analysis

Stratified analyses by BMI Stratified linear regression models revealed distinct UA-BMD associations across BMI categories.

Subsequent stratified analysis confirmed that the positive association between UA and BMD remained robust specifically within the normal BMI subgroup (18.5–24.9 $kg/m^{2},R^{2}=0.05,P=0.01$). Conversely, this relationship was not observed in either the underweight ( $<18.5\;kg/m^{2}$) or overweight/obese $(\geq 25\;kg/m^{2}$) cohorts. Specifically, in the high BMI group, the regression coefficient failed to reach significance ( P=0.79) with negligible explanatory power ( $R^{2}=0.001$). These findings suggest that BMI acts as a key effect modifier, with the positive UA-BMD correlation being primarily confined to the normal-weight population (**Figure 3**).

### Stratified analyses by calcium and creatinine

Stratified analyses further elucidated how calcium and renal status modify the UA-BMD relationship.

#### Calcium subgroups

In the low-calcium subgroup (<2.1 mmol/L), UA showed no significant association with BMD (R^2^ = 0.020, P = 0.645). However, in the normal calcium subgroup (2.1-2.5 mmol/L), a marginally significant positive trend was observed (R^2^ = 0.017, P = 0.067). These findings suggest that adequate calcium levels may be a prerequisite for the potential positive influence of UA on bone density.

#### Creatinine subgroups

The association between UA and BMD varied notably across creatinine levels. In the low creatinine group, UA explained a relatively higher proportion of BMD variability (R^2^ = 0.091) and demonstrated a significant positive association (P = 0.014).

**Figure 4:**
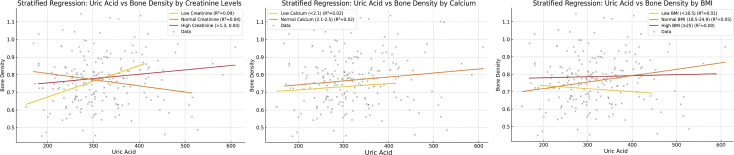
Variation in the association between serum uric acid and bone mineral density (BMD) across different serum creatinine level groups.

However, this protective effect attenuated with rising creatinine levels. In the normal creatinine group, the association weakened and approached marginal significance (R^2^ = 0.041, P = 0.076), with the trend shifting toward a negative correlation. Finally, in the high creatinine group, the explanatory power was minimal (R^2^ = 0.032), and no significant association was found (P = 0.129) (**Figure 4**).

### Feature importance analysis

**Figure 5:**
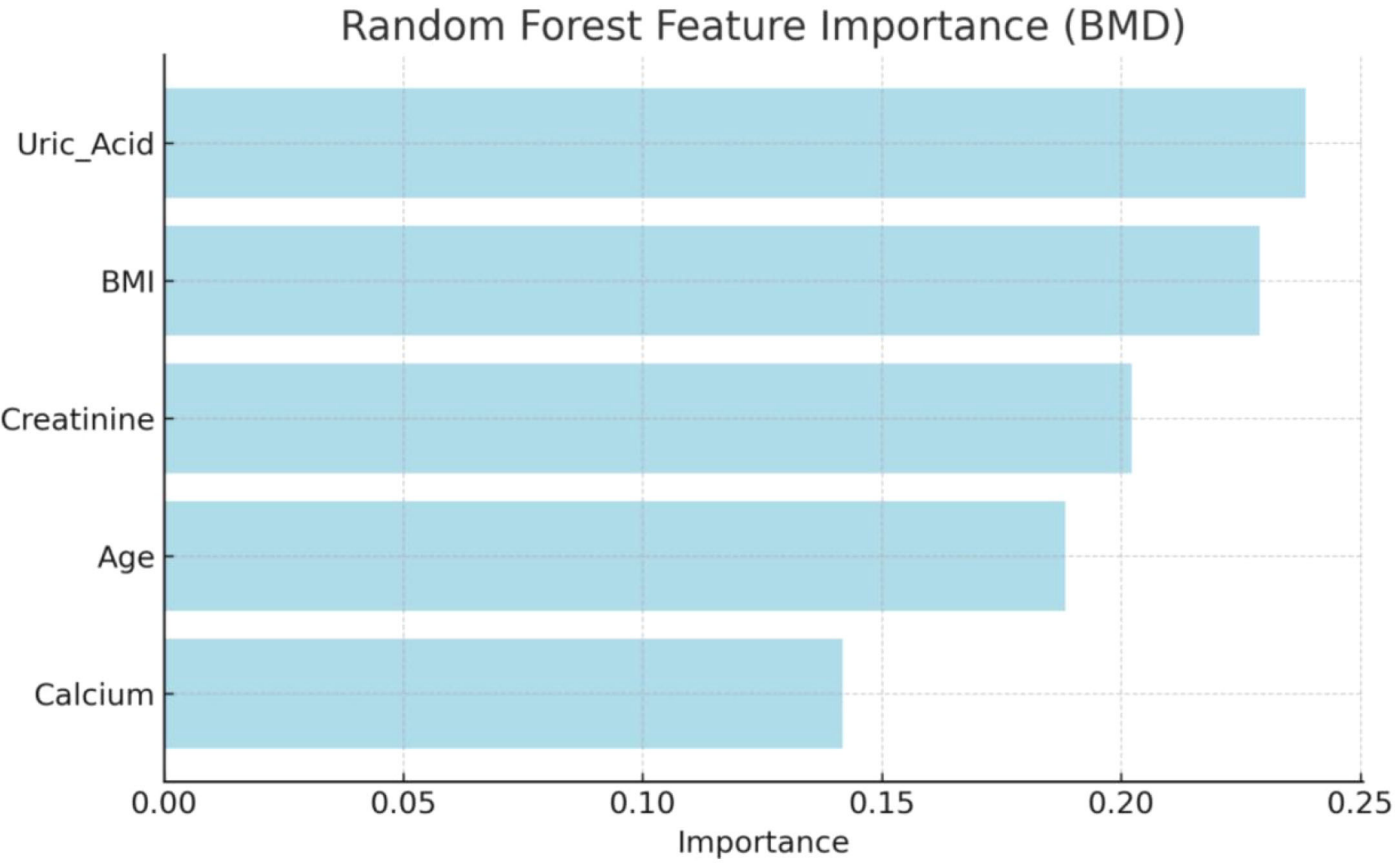
Feature importance analysis quantifies the relative contribution of each variable to bone mineral density (BMD).

Feature importance analysis to quantify the relative contribution of each variable to BMD, a feature importance analysis was conducted using a random forest algorithm (**Figure 5**). UA emerged as the most influential predictor, yielding the highest importance score of 0.2386. This was closely followed by BMI (0.2290) and serum creatinine (0.2022), which also demonstrated substantial predictive value. In comparison, age (0.1884) and serum calcium (0.1418) exhibited moderate contributions. Collectively, these analyses highlight UA and BMI as the primary predictive factors within the model.

## Discussion

### Role of uric acid in bone health

In this cohort of adults aged over 50 years, UA demonstrated a positive but non-linear association with BMD, consistent with prior studies reporting similar correlations in older adults and osteoporotic patients (19, 20): BMD increased with UA at lower concentrations (≤200 μmol/L) but plateaued at levels exceeding 400 μmol/L, suggesting a saturation effect rather than a sustained dose-dependent relationship. Several mechanisms may underlie this protective effect, including UA’s role as a major antioxidant in human plasma (accounting for approximately 60% of free radical scavenging capacity (20), its capacity to promote bone formation via the MAPK signaling pathway, and its inhibition of RANKL-induced osteoclastogenesis (21).These findings carry potential clinical implications, as UA levels are modifiable through dietary or pharmacological approaches; nevertheless, any strategy targeting UA elevation must be carefully weighed against associated risks, including gout, cardiovascular disease, and chronic kidney disease.

### Impact of BMI on BMD

BMI exhibited a characteristic inverted U-shaped association with BMD in our cohort. Within the normal BMI range (18.5–24.9kg/m²), increases in body weight were positively correlated with BMD, likely due to enhanced mechanical loading that stimulates bone formation (20, 21). However, once BMI exceeded 25 kg/m²,the protective effect waned, potentially owing to obesity-related factors such as metabolic syndrome, chronic inflammation, and adipose tissue interference with bone metabolism. This is supported by research in diverse populations, where the UA-BMD relationship was stronger in normal-weight individuals, underscoring the role of BMI as a critical modifier (20, 22). For example, in.

middle-aged and elderly men, a positive association between UA and BMD was only evident in the normal BMI group, emphasizing the need to consider weight status in bone health assessments (21).

### Dual role of creatinine

Creatinine levels displayed a dual influence on BMD in our study. At lower concentrations (<80 μmol/L), creatinine was associated with a significant positive effect on BMD, possibly reflecting higher muscle mass, which is known to promote bone strength through mechanical stimuli (20). However, as creatinine levels rose above 100 μmol/L, this beneficial effect plateaued or declined, likely due to its correlation with renal impairment. Kidney dysfunction can lead to disturbances in calcium and phosphorus homeostasis, adversely affecting bone density (20). Although direct evidence on creatinine and BMD is limited in the literature, studies incorporating renal parameters like blood urea nitrogen have shown similar trends, reinforcing the idea that renal health is integral to bone metabolism (20).

### Independent effects of age and calcium

Age emerged as a strong independent negative predictor of BMD, with a notable acceleration in bone loss after 65 years, consistent with physiological aging processes such as reduced estrogen levels and increased bone resorption (19, 23). For instance, research in elderly women with vertebral compression fractures highlighted age-dependent variations in the UA-BMD relationship, underscoring the heightened vulnerability in older subgroups (23).Regarding calcium, levels within the normal range (2.1–2.5 mmol/L) were positively associated with BMD, likely due to calcium’s crucial role in bone mineralization. However, beyond 2.5 mmol/L, the effect stabilized, suggesting that excessive calcium may not confer additional benefits and could even be counterproductive if not balanced with other nutrients. This aligns with studies in postmenopausal women, where maintaining normal calcium levels was pivotal for osteoporosis prevention (24).

### Interactions and variable importance

Interaction analyses revealed significant effects between UA and BMI, as well as UA and creatinine, on BMD. For example, the protective role of UA was more pronounced in normal BMI ranges, whereas high creatinine levels mitigated its positive impact, indicating that metabolic and renal factors interact to shape bone health outcomes. These findings are corroborated by subgroup analyses in previous studies, such as those stratified by BMI or race, which demonstrated modified associations (19, 20). Furthermore, random forest importance analysis identified creatinine (25.96%), UA (23.50%), and BMI(20.97%) as the most influential variables affecting BMD, highlighting the centrality of muscle mass (reflected by creatinine), antioxidant capacity (linked to UA), and weight management in maintaining bone density. This multivariate approach echoes the complexity observed in epidemiological research, where factors like age, gender, and renal function often confound the UA-BMD relationship (20, 21, 25).In conclusion, our results emphasize the non-linear and interactive nature of UA, BMI, creatinine, age, and calcium in influencing BMD among older adults. These insights advocate for personalized bone health strategies that account for individual metabolic and renal profiles, while also pointing to the need for longitudinal studies to establish causality.

### Limitations and future directions

While this study provides valuable insights, several limitations should be acknowledged. The cross-sectional design precludes causal inference, and residual confounding may persist despite comprehensive multivariable adjustment. The study population consisted of adults over 50 years from specific geographic regions, which may limit generalizability to other populations. Future research should include longitudinal assessments to establish temporal relationships, more detailed body composition measures using advanced imaging techniques, and investigation of specific inflammatory markers and hormonal mediators that might explain the observed relationships. Additional studies are needed to elucidate the molecular mechanisms underlying the biphasic effect of uric acid on bone metabolism and to determine optimal UA levels for bone health in different population subgroups. Furthermore, intervention studies targeting uric acid modulation, weight management, and renal function preservation could provide direct evidence for causal relationships and inform clinical practice guidelines for osteoporosis prevention and management in aging populations.

## Data Availability

The original contributions presented in the study are included in the article/supplementary material. Further inquiries can be directed to the corresponding author.

## References

[1] Luo J Li L Shi W Xu K Shen Y Dai B . Oxidative stress and inflammation: roles in osteoporosis. Front Immunol. (2025) 16:1611932. doi: 10.3389/fimmu.2025.1611932, PMID: 40873591 PMC12379731

[2] Wimalawansa SJ . Vitamin D deficiency: effects on oxidative stress, epigenetics, gene regulation, and aging. Biol (Basel). (2019) 8:30. doi: 10.3390/biology8020030, PMID: 31083546 PMC6627346

[3] Kimball JS Johnson JP Carlson DA . Oxidative stress and osteoporosis. J Bone Joint Surg Am. (2021) 103:1451–61. doi: 10.2106/JBJS.20.00989, PMID: 34014853

[4] Iantomasi T Romagnoli C Palmini G Donati S Falsetti I Miglietta F . Oxidative stress and inflammation in osteoporosis: molecular mechanisms involved and the relationship with microRNAs. Int J Mol Sci. (2023) 24:3772. doi: 10.3390/ijms24043772, PMID: 36835184 PMC9963528

[5] Li X Peng Y Chen K Zhou Y Luo W . Association between serum uric acid levels and bone mineral density in Chinese and American: a cross-sectional study. Sci Rep. (2025) 15:8304. doi: 10.1038/s41598-025-92348-3, PMID: 40064963 PMC11894223

[6] Ando A Mitsuhashi T Honda M Hanayama Y Hasegawa K Obika M . Risk factors for low bone mineral density determined in patients in a general practice setting. Acta Med Okayama. (2019) 73:403–11. doi: 10.18926/AMO/57370, PMID: 31649366

[7] Cong B Zhang H . The effects of combined calcium and vitamin D supplementation on bone mineral density and fracture risk in postmenopausal women with osteoporosis: a systematic review and meta-analysis of randomized controlled trials. BMC Musculoskelet Disord. (2025) 26:928. doi: 10.1186/s12891-025-09089-7, PMID: 41063100 PMC12506016

[8] Bhattarai HK Shrestha S Rokka K Shakya R . Vitamin D, calcium, parathyroid hormone, and sex steroids in bone health and effects of aging. J Osteoporos. (2020) 2020:9324505. doi: 10.1155/2020/9324505, PMID: 32612801 PMC7317615

[9] Voulgaridou G Papadopoulou SK Detopoulou P Tsoumana D Giaginis C Kondyli FS . Vitamin D and calcium in osteoporosis, and the role of bone turnover markers: A narrative review of recent data from RCTs. Diseases. (2023) 11:29. doi: 10.3390/diseases11010029, PMID: 36810543 PMC9944083

[10] Migliorini F Maffulli N Colarossi G Filippelli A Memminger M Conti V . Vitamin D and calcium supplementation in women undergoing pharmacological management for postmenopausal osteoporosis: a level I of evidence systematic review. Eur J Med Res. (2025) 30:170. doi: 10.1186/s40001-025-02412-x, PMID: 40087804 PMC11907966

[11] Jiao D Jiang C . Nutritional therapy of older osteoporotic people with supplemental calcium and vitamin D: side effects, fracture rates, and survival - an internationalised meta-analysis. Asia Pac J Clin Nutr. (2024) 33:1–10. doi: 10.6133/apjcn.202403_33(1).0001, PMID: 38494682 PMC11170020

[12] Hasegawa T Hongo H Yamamoto T Abe M Yoshino H Haraguchi-Kitakamae M . Matrix vesicle-mediated mineralization and osteocytic regulation of bone mineralization. Int J Mol Sci. (2022) 23:9941. doi: 10.3390/ijms23179941, PMID: 36077336 PMC9456179

[13] Bourne LE Wheeler-Jones CP Orriss IR . Regulation of mineralisation in bone and vascular tissue: a comparative review. J Endocrinol. (2021) 248:R51–65. doi: 10.1530/JOE-20-0428, PMID: 33337345

[14] Gómez-De-Tejada-Romero MJ Murias-Henríquez C Saavedra-Santana P Sablón-González N Abreu DR Sosa-Henríquez M . Influence of serum uric acid on bone and fracture risk in postmenopausal women. Aging Clin Exp Res. (2024) 36:156. doi: 10.1007/s40520-024-02819-2, PMID: 39085733 PMC11291523

[15] Malagrinò M Zavatta G . Uric acid in primary hyperparathyroidism: marker, consequence, or bystander? Metabolites. (2025) 15:444. doi: 10.3390/metabo15070444, PMID: 40710544 PMC12300645

[16] Wu H Li H Dai X Dai Y Liu H Xu S . A Mendelian randomization study of the association between serum uric acid and osteoporosis risk. Front Endocrinol (Laus). (2024) 15:1434602. doi: 10.3389/fendo.2024.1434602, PMID: 39464184 PMC11502378

[17] Huang T He Y Li Y Zhang H Wang Q Gao Y . The relationship between serum fibroblast growth factor 23 and klotho protein and low bone mineral density in middle-aged and elderly patients with end-stage renal disease. Horm Metab Res. (2024) 56:142–9. doi: 10.1055/a-2168-5089, PMID: 37875141

[18] Guan Y Lu YH Lei SF . Physical activities mediate the correlations between serum creatinine and bone mineral density in Chinese. Clin Chim Acta. (2022) 524:25–33. doi: 10.1016/j.cca.2021.11.025, PMID: 34843706

[19] Yao X Chen L Xu H Zhu Z . The association between serum uric acid and bone mineral density in older adults. Int J Endocrinol. (2020) 2020:3082318. doi: 10.1155/2020/3082318, PMID: 32676109 PMC7341403

[20] Xu MZ Lu K Yang XF Ye YW Xu SM Shi Q . Association between serum uric acid levels and bone mineral density in patients with osteoporosis: a cross-sectional study. BMC Musculoskelet Disord. (2023) 24:306. doi: 10.1186/s12891-023-06414-w, PMID: 37072779 PMC10111842

[21] Lin ZC Wu JF Chang CY Lai KM Yang HY . Association between serum uric acid level and bone mineral density at multiple skeletal sites in middle-aged and elderly men: a cross-sectional study of a healthy population in Taiwan. Arch Osteoporos. (2022) 17:142. doi: 10.1007/s11657-022-01186-7, PMID: 36376511

[22] Ibrahim WN Younes N Shi Z Abu-Madi MA . Serum uric acid level is positively associated with higher bone mineral density at multiple skeletal sites among healthy Qataris. Front Endocrinol (Laus). (2021) 12:653685. doi: 10.3389/fendo.2021.653685, PMID: 33868180 PMC8044437

[23] Zhu J Xia Z Min J Hu W Li H Mei C . Age-stratified association between serum uric acid and lumbar bone mineral density in elderly Chinese women with vertebral compression fractures: a cross-sectional analysis. Front Med (Laus). (2025) 12:1591791. doi: 10.3389/fmed.2025.1591791, PMID: 40978736 PMC12446221

[24] Tu J Mo X Zhang X Chen Z Xi L Wu C . BMI mediates the association of serum uric acid with bone health: a cross-sectional study of the National Health and Nutrition Examination Survey (NHANES). BMC Musculoskelet Disord. (2024) 25:482. doi: 10.1186/s12891-024-07595-8, PMID: 38898434 PMC11186245

[25] Li X Li L Yang L Yang J Lu H . No association between serum uric acid and lumbar spine bone mineral density in US adult males: a cross sectional study. Sci Rep. (2021) 11:15588. 34341438 10.1038/s41598-021-95207-z PMC8329127

